# Phylogenetic analysis of complete genome sequences of hepatitis B virus from an Afro-Colombian community: presence of HBV F3/A1 recombinant strain

**DOI:** 10.1186/1743-422X-9-244

**Published:** 2012-10-24

**Authors:** Mónica V Alvarado-Mora, Camila M Romano, Michele S Gomes-Gouvêa, Maria F Gutierrez, Flair J Carrilho, João R R Pinho

**Affiliations:** 1 Laboratory of Gastroenterology and Hepatology, São Paulo Institute of Tropical Medicine and Department of Gastroenterology, School of Medicine, University of São Paulo, São Paulo, Brazil; 2 São Paulo Institute of Tropical Medicine, Department of Infectious and Parasitic Diseases (LIMHC), School of Medicine, University of São Paulo, São Paulo, Brazil; 3 Laboratory of Virology, Department of Microbiology, Pontificia Javeriana University, Bogotá, Colombia

**Keywords:** Hepatitis B virus, Subgenotype A1, Genotype E, Subgenotype D3, Bayesian analysis, Recombinant subgenotype F3/A1, Colombia

## Abstract

**Background:**

Hepatitis B virus (HBV) infection is one of the most prevalent viral infections in humans and represents a serious public health problem. In Colombia, our group reported recently the presence of subgenotypes F3, A2 and genotype G in Bogotá. The aim of this study was to characterize the HBV genotypes circulating in Quibdó, the largest Afro-descendant community in Colombia. Sixty HBsAg-positive samples were studied. A fragment of 1306 bp (S/POL) was amplified by nested PCR. Positive samples to S/POL fragment were submitted to PCR amplification of the HBV complete genome.

**Findings:**

The distribution of HBV genotypes was: A1 (52.17%), E (39.13%), D3 (4.3%) and F3/A1 (4.3%). An HBV recombinant strain subgenotype F3/A1 was found for the first time.

**Conclusions:**

This study is the first analysis of complete HBV genome sequences from Afro-Colombian population. It was found an important presence of HBV/A1 and HBV/E genotypes. A new recombinant strain of HBV genotype F3/A1 was reported in this population. This fact may be correlated with the introduction of these genotypes in the times of slavery.

## Introduction

Hepatitis B virus (HBV) infection is a relevant global health problem with 2 billion people that have been infected worldwide, including 350 million of them suffering from chronic HBV infection [1]. In Latin America, the estimated HBsAg seroprevalence ranges from 0.5% to 3.0%, with the total number of HBsAg carriers approaching 11 millions [2]. The highest prevalence surpassing 8.0 % is found among the native populations of the western Amazon basin, which includes Brazil [3], Colombia [4], Peru [5] and Venezuela [6].

HBV genome is a partially double-stranded circular DNA molecule of approximately 3,200 bp that encodes four overlapping open reading frames (ORFs) [7]. A genetic classification based on the comparison of complete HBV genomes has identified nine genotypes, A through I [8], that differ by at least 8% at nucleotide level from each other. Genotype A was initially identified in southern Africa [9]. Phylogenetic analysis of the complete genomes of subgenotype A1 isolates classified it in two clusters (African and Asian) [10]. The introduction of subgenotype A1 into Asia could be the result of movements along the East coast of Africa, from Somalia in the horn of Africa to the Arabic Peninsula in Asia [10]. Subgenotype A2, also denoted Ae, from “European” subgenotype, was isolated from South African carriers and is also found in Northern Europe and Greenland [11]. Subsequently, subgenotype A3 was characterized in Cameroon, Mali and Gambia [12]. Complete genomes of two HBV isolates from Mali (subgenotype A4) have been found to diverge by 6% from A3. Finally, four Nigerian genotype A isolates formed another group, tentatively designated subgenotype A5 [13]. More recently, subgenotypes A6 was detected in African-Belgian patients [14] and subgenotype A7 was reported in Cameroon [15].

Genotypes B and C are predominant in East and Southeast Asia. Subgenotype D1 occurs mostly in the Mediterranean basin and Middle East. D2 has been reported in India, Japan, Europe and the United States. D3 was found in South Africa, Brazil, Rwanda, India, Costa Rica, Iran, Serbia and the United States. Finally, D4 was reported in Australia, South Africa, Somalia, Rwanda and Oceania [11].

Genotype E was first described in West Africa in high prevalence but in a surprisingly low diversity, as the mean diversity over the whole genome is 1.75%. HBV/E is the most prevalent genotype in western and central Africa [10]. In Quibdó, Colombia we previously reported for the fist time the presence of this genotype in nine cases [16]. Genotypes F and H are found in populations from Alaska to Central and South America [17,18].

The aim of the present study was to characterize the HBV genotypes circulating in Quibdó city, Colombia. We report for the first time the HBV complete genome sequences from Afro-Colombian infected people and inferred their origin using phylogenetic analyses approaches.

## Materials and methods

### Study population

To evaluate HBV genotypes distribution in Quibdó, the largest Afro-descendant community in Colombia, 60 positive samples for the Hepatitis B virus surface antigen (HBsAg) were obtained from sera stored at −20°C in a public health laboratory in Quibdó, Colombia in 2007. This protocol was approved by the Ethical Committees from Pontificia Universidad Javeriana, Bogotá Colombia and School of Medicine, University of São Paulo, São Paulo, Brazil. Before

### HBV DNA extraction

HBV DNA extraction was carried out from 100 μl serum using the acid guanidinium thiocyanate/phenol/chloroform method [19]. Briefly, 300 μL GT solution was added to each sample. Ice-cold chloroform (50 μL) was added, followed by homogenization and centrifugation. The supernatant was transferred to a conical tube and precipitated with 300 μL cold ethanol. After discarding the ethanol, samples were dried at 94**°**C for 1 min, resuspended in 50 μL ultrapure MilliQ water and stored at −20**°**C.

### HBV PCR amplification

To characterize HBV genotypes, a fragment of 1306 bp partially comprising HBsAg and DNA polymerase coding regions (S/POL) was amplified by nested PCR using the primers PS3132F/2920R and PS3201F/P1285R [18]. After purification of the PCR product through ChargeSwitch PCR Clean-Up Kit, Sanger sequencing was performed using dideoxynucleotide triphosphates (ddNTPs) in Big Dye Terminator v3.1 Cycle Sequencing Ready Reaction kit – Applied Biosystems, Foster City, CA, USA). The electrophoresis was done in an ABI Prism 377 Automatic Sequencer (Applied Biosystems, Foster City, CA, USA). The quality of each electropherogram was evaluated using the Phred-Phrap software and consensus sequences were obtained by alignment of both sequenced strands (sense and antisense) using CAP3 software available at the web page Electropherogram quality analysis Phred (http://asparagin.cenargen.embrapa.br/phph).

Amplification of the whole HBV genome was performed with P1 and P2 primers described previously with slight modifications [20]. The quality of each electropherogram was evaluated as cited above.

### HBV genotyping analysis

Sequences were genotyped by phylogenetic reconstructions using reference sequences from all HBV genotypes obtained from GenBank (n=412), comprising 1306 bp of partial HBsAg and DNA polymerase coding regions (S/POL). Complete genomes where also obtained from Genbank (n=192) and phylogenetic analyses were performed. All sequences were aligned using Muscle software [21] and edited with the SE-AL software (available at http://tree.bio.ed.ac.uk/software/seal/). The Bayesian Markov chain Monte Carlo (MCMC) simulation implemented in BEAST v.1.5.4 [22] was done to obtain the best possible estimates under both relaxed uncorrelated log_normal_ and exponential molecular clock and using the model of nucleotide substitution (GTR+G+I). The molecular clock that best fitted the data was chosen by Bayes factor (BF) comparison. After 10 million generations, the maximum credibility tree (MCC) was obtained by summarizing the 10,000 substitution trees after removing a burn-in of 10% using Tree Annotator v.1.5.3 [22]. Phylogenetic trees were visualized and midpoint rooted in FigTree v1.2.2 (http://tree.bio.ed.ac.uk/software/figtree/).

## Results

**Figure 1:**
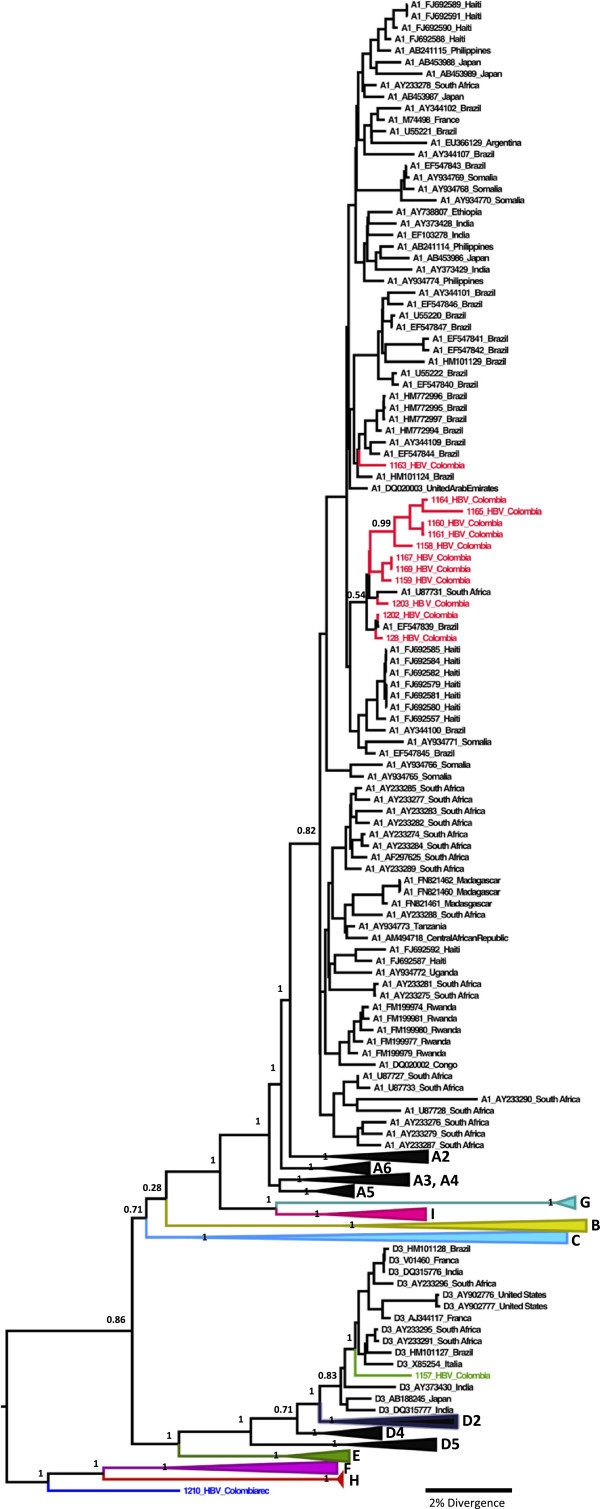
**The Maximum Clade Credibility (MCC) tree was estimated by a Bayesian analysis from a larger dataset comprising 413 sequences with 1306 nucleotides of S and Polymerase HBV region of the different subgenotypes.** The posterior probabilities of the key nodes are show above the respective clusters. The cluster containing the strains of other HBV subgenotypes were collapsed.

Of the 60 HbsAg-positive samples, 29 (48.3%) were positive by nested PCR for S/POL region and among them, 23 were obtained with good quality sequenced for phylogenetic analysis (Figure 1). The distribution of HBV genotypes in these 23 samples was: A1 (52.17%), E (39.13%), D3 (4.3%) and F3/A1 (4.3%). Nine genotype E sequences generated in this study from the same community were previously published [16] since they represented an exclusively African HBV genotype circulating in South America. Due to a small sample size, these results were considered inconclusive about the prevalence of these genotypes in the population.

**Figure 2:**
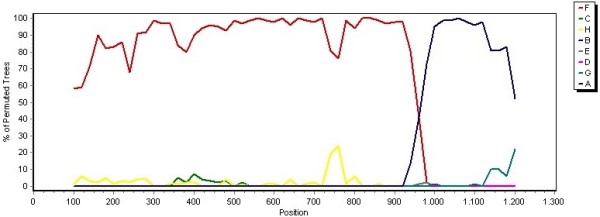
**Nucleotide similarity comparisons with consensus sequences represented each HBV genotype (A-I) and the Colombian recombinant strain HBV F3/A1.** The break point of the Colombian strain is at position 941 of HBV genome (POL region).

**Figure 3:**
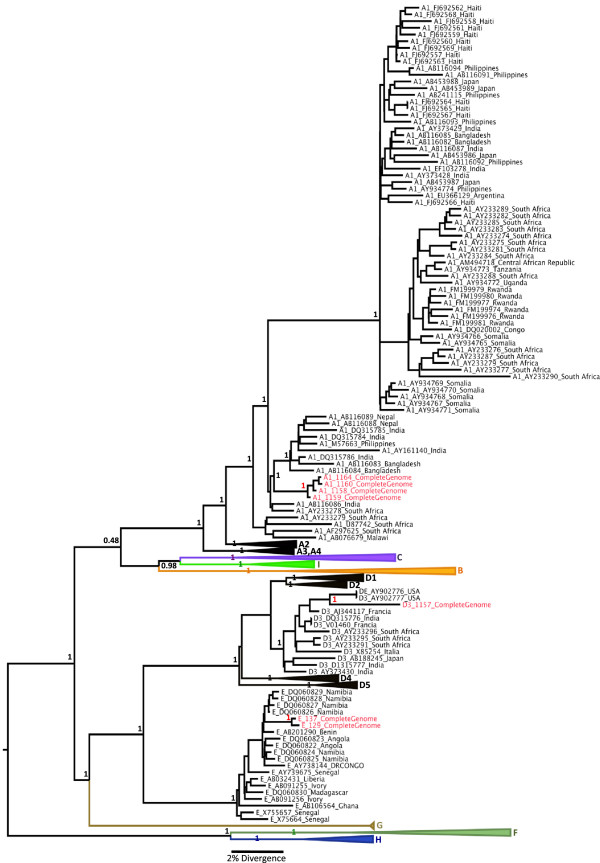
**The Maximum Clade Credibility (MCC) tree was estimated by a Bayesian analysis of 192 complete genome sequences of HBV strains.** The posterior probabilities of the key nodes are shown above the respective nodes. The HBV Colombian complete genome sequences (n = 7) were analyzed together with other strains from around the world. The cluster containing the strains of other HBV subgenotypes were collapsed.

This work shows for the first time globally a recombinant strain of HBV genotype F3/A1 with 1306bp. The break point was at position 941 of HBV genome (POL region). The analysis using the Simplot program and bootscanning analysis confirmed this complex recombination. The sequence was compared with a consensus sequence of each HBV genotype (A–H) in order to identify the breakpoints. The analysis was carried out using a window size of 200 bp, a step size of 20 bp, 100 bootstrap replicates, gapstrip on and neighbour-joining analysis. The break point at position 941 of HBV genome (POL region) was in the codon ATT (C) (HBV/F3) → ACT (C) (HBV/A1) (Figure 2). Furthermore, we obtained for the first time seven HBV Colombian complete genome sequences from A1, E and D3 genotypes (Figure 3). Complete genome HBV sequences were deposited at the GenBank under accession numbers: JQ023660-JQ023666.

## Discussion

These results also showed that the distribution pattern of HBV genotypes and/or genotypes variation could be different in Colombia based on the region studied since unlike in Quibdó, a previous study in Bogota found that the most frequent subgenotype was the subgenotype F3 followed by A2 and G [18]. The presence of genotype E in more than two thirds of cases studied herein highlights the importance to carry out larger studies in Quibdó population to ascertain if this genotype is widespread in this region.

Such results could provide additional knowledge on the history of HBV/E around the world and further clarify molecular chronometer of such viral infections among humans. Based on our recent publication on genotype E in this afro-Colombian community [16], the TMRCA data suggested a recent infection; however such high frequency of infection in a population does not seem to match a recent infection. Further investigations are required to correlate these findings.

In Africa, viruses belonging to five genotypes, A (HBV/A) to E (HBV/E), have been found. Subgenotype A1 was identified in HBV isolates from South Africa using phylogenetic analysis of preS2/S sequences and confirmed by analysis of complete genomes from South Africa and Malawi [9]. This subgenotype has also been found in Somalia, Zimbabwe, Kenya, Rwanda, Philippines, India and Nepal and Yemen [10]. In South America, previous studies have shown that subgenotype A1 was the most frequent in Brazilian population [23]. While in Argentina there is a low prevalence of this subgenotype [24], in Haiti more than 90% of the population descended from African Slaves has the A1 as the most common subgenotype [25]. While this suggest that subgenotype A1 in South America is probably of similar origin, the subgenotype A1 in Colombia reported in this study though grouped in the same cluster, seems so suggest otherwise since the posterior probability was low (0.21), thus indicating a distant genetic relatedness.

An important result of this work was the presence of recombinant strain between subgenotype F3 and A1 found in a sixteen years old girl. Since this girl was pregnant when this sample was collected, is probably that sexual contact was the way of HBV transmission. Several HBV recombinants inter genotypes have been reported around the world. In Bolivia, South America, several recombinants A/D, C/B, D/C and F/C have been detected [26]. Furthermore, recombination within and between genotypes created complex patterns and altered the cladistic structure of HBV genotypes [27]. For example, in previous study the B2 subgenotype proved to be a hybrid of genotypes B and C [28]. Unfortunately, we had no success on full-length genome amplification of our recombinant strain. Thus, although we know that the break point was in the polymerase region, we cannot exclude the possibility with of existence of other recombination points in this virus. In sum, this is the first HBV recombinant strain reported in Colombia.

Here, we present a study of the first seven complete HBV genomes of Colombian population. The results obtained from E and A1 genotypes support the theory that HBV may have been was introduced into this Afro-descendent community in Colombia in the times of slavery.

## Competing interests

The authors declare that they have no competing interests.

## Author’s contributions

MVAM participated in the design of the study and drafted the manuscript, conducted the sequencing process and phylogenetic analysis. CMR conducted the recombination and evolutionary analysis. MSGG participated in the PCR amplification. MFG and FJC participated in the design of the study. JRRP participated in the elaboration of the manuscript. All authors read and approved the final manuscript.

## References

[B1] Lee WM Hepatitis B virus infection N Engl J Med 1997 337 24 1733 1745 10.1056/NEJM199712113372406 9392700

[B2] Tanaka J Hepatitis B epidemiology in Latin America Vaccine 2000 18 Suppl 1 S17 S19 10683537 10.1016/s0264-410x(99)00455-7

[B3] Arboleda M Castilho MC Fonseca JC Albuquerque BC Saboia RC Yoshida CF Epidemiological aspects of hepatitis B and D virus infection in the northern region of Amazonas, Brazil Trans R Soc Trop Med Hyg 1995 89 481 483 10.1016/0035-9203(95)90074-8 8560515

[B4] Alvarado-Mora MV Fernandez MF Gomes-Gouvea MS de Azevedo Neto RS Carrilho FJ Pinho JR Hepatitis B (HBV), hepatitis C (HCV) and hepatitis delta (HDV) viruses in the Colombian population--how is the epidemiological situation? PLoS One 2011 6 4 e18888 10.1371/journal.pone.0018888 21559488 PMC3084727

[B5] Fay OH Hepatitis B in Latin America: epidemiological patterns and eradication strategy. The Latin American Regional Study Group Vaccine 1990 8 S100 S106 2139277

[B6] Duarte MC Cardona N Poblete F Gonzalez K Garcia M Pacheco M Botto C Pujol FH Williams JR A comparative epidemiological study of hepatitis B and hepatitis D virus infections in Yanomami and Piaroa Amerindians of Amazonas State, Venezuela Trop Med Int Health 2010 15 8 924 933 10.1111/j.1365-3156.2010.02560.x 20561309

[B7] Stuyver L De Gendt S Van Geyt C Zoulim F Fried M Schinazi RF Rossau R A new genotype of hepatitis B virus: complete genome and phylogenetic relatedness J Gen Virol 2000 81 Pt 1 67 74 10640543 10.1099/0022-1317-81-1-67

[B8] Yu H Yuan Q Ge SX Wang HY Zhang YL Chen QR Zhang J Chen PJ Xia NS Molecular and phylogenetic analyses suggest an additional hepatitis B virus genotype "I" PLoS One 2010 5 2 e9297 10.1371/journal.pone.0009297 20174575 PMC2824819

[B9] Kramvis A Weitzmann L Owiredu WK Kew MC Analysis of the complete genome of subgroup A' hepatitis B virus isolates from South Africa J Gen Virol 2002 83 Pt 4 835 839 11907333 10.1099/0022-1317-83-4-835

[B10] Kramvis A Kew MC Molecular characterization of subgenotype A1 (subgroup Aa) of hepatitis B virus Hepatol Res 2007 37 s1 S27 S32 10.1111/j.1872-034X.2007.00100.x 17627631

[B11] Norder H Courouce AM Coursaget P Echevarria JM Lee SD Mushahwar IK Robertson BH Locarnini S Magnius LO Genetic diversity of hepatitis B virus strains derived worldwide: genotypes, subgenotypes, and HBsAg subtypes Intervirology 2004 47 6 289 309 10.1159/000080872 15564741

[B12] Hannoun C Soderstrom A Norkrans G Lindh M Phylogeny of African complete genomes reveals a West African genotype A subtype of hepatitis B virus and relatedness between Somali and Asian A1 sequences J Gen Virol 2005 86 Pt 8 2163 2167 16033963 10.1099/vir.0.80972-0

[B13] Olinger CM Venard V Njayou M Oyefolu AO Maiga I Kemp AJ Omilabu SA le Faou A Muller CP Phylogenetic analysis of the precore/core gene of hepatitis B virus genotypes E and A in West Africa: new subtypes, mixed infections and recombinations J Gen Virol 2006 87 Pt 5 1163 1173 16603517 10.1099/vir.0.81614-0

[B14] Pourkarim MR Lemey P Amini-Bavil-Olyaee S Maes P Van Ranst M Novel hepatitis B virus subgenotype A6 in African-Belgian patients J Clin Virol 2010 47 1 93 96 10.1016/j.jcv.2009.09.032 19857994

[B15] Hubschen JM Mbah PO Forbi JC Otegbayo JA Olinger CM Charpentier E Muller CP Detection of a new subgenotype of hepatitis B virus genotype A in Cameroon but not in neighbouring Nigeria Clin Microbiol Infect 2011 17 1 88 94 10.1111/j.1469-0691.2010.03205.x 20219082

[B16] Alvarado Mora MV Romano CM Gomes-Gouvea MS Gutierrez MF Carrilho FJ Pinho JR Molecular epidemiology and genetic diversity of hepatitis B virus genotype E in an isolated Afro-Colombian community J Gen Virol 2010 91 Pt 2 501 508 19846674 10.1099/vir.0.015958-0

[B17] Devesa M Loureiro CL Rivas Y Monsalve F Cardona N Duarte MC Poblete F Gutierrez MF Botto C Pujol FH Subgenotype diversity of hepatitis B virus American genotype F in Amerindians from Venezuela and the general population of Colombia J Med Virol 2008 80 1 20 26 10.1002/jmv.21024 18041024

[B18] Alvarado Mora MV Romano CM Gomes-Gouvea MS Gutierrez MF Botelho L Carrilho FJ Pinho JR Molecular characterization of the Hepatitis B virus genotypes in Colombia: a Bayesian inference on the genotype F Infect Genet Evol 2011 11 1 103 108 10.1016/j.meegid.2010.10.003 20951841

[B19] Chomczynski P Sacchi N The single-step method of RNA isolation by acid guanidinium thiocyanate-phenol-chloroform extraction: twenty- something years on Nat Protoc 2006 1 2 581 585 10.1038/nprot.2006.83 17406285

[B20] Gomes-Gouvea MS Characterization of complete genomes of the hepatitis B virus of different genotypes isolated in Brazil 2005 São Paulo: Secretaria da Saúde/Coordenadoria de Controle de Doenças. Programa de Pós-Graduação em Ciências para obtenção do grau de Mestre 130

[B21] Edgar RC MUSCLE: a multiple sequence alignment method with reduced time and space complexity BMC Bioinformatics 2004 5 113 10.1186/1471-2105-5-113 15318951 PMC517706

[B22] Drummond AJ Rambaut A BEAST: Bayesian evolutionary analysis by sampling trees BMC Evol Biol 2007 7 214 10.1186/1471-2148-7-214 17996036 PMC2247476

[B23] Motta-Castro AR Martins RM Yoshida CF Teles SA Paniago AM Lima KM Gomes SA Hepatitis B virus infection in isolated Afro-Brazilian communities J Med Virol 2005 77 2 188 193 10.1002/jmv.20435 16121385

[B24] Mbayed VA Pineiro y Leone FG Pezzano SC Campos RH Molecular characterization of hepatitis B virus genotype A from Argentina and Brazil Arch Virol 2009 154 3 525 529 10.1007/s00705-009-0328-6 19225714

[B25] Andernach IE Nolte C Pape JW Muller CP Slave trade and hepatitis B virus genotypes and subgenotypes in Haiti and Africa Emerg Infect Dis 2009 15 8 1222 1228 10.3201/eid1508.081642 19751583 PMC3467954

[B26] Simmonds P Midgley S Recombination in the genesis and evolution of hepatitis B virus genotypes J Virol 2005 79 24 15467 15476 10.1128/JVI.79.24.15467-15476.2005 16306618 PMC1316029

[B27] Purdy MA Gonzales AC Dimitrova Z Khudyakov Y Supragenotypic groups of the hepatitis B virus genome J Gen Virol 2008 89 Pt 5 1179 1183 18420795 10.1099/vir.0.83392-0

[B28] Sugauchi F Orito E Ichida T Kato H Sakugawa H Kakumu S Ishida Chutaputti A Lai CL Ueda R Hepatitis B virus of genotype B with or without recombination with genotype C over the precore region plus the core gene J Virol 2002 76 12 5985 5992 10.1128/JVI.76.12.5985-5992.2002 12021331 PMC136227

